# PDBTools.jl: A Lightweight and High-Performance Julia Package for Molecular Structure File Handling and Analysis

**DOI:** 10.1021/acs.jcim.6c00847

**Published:** 2026-05-19

**Authors:** Leandro Martínez, Ana B. B. Lima

**Affiliations:** Institute of Chemistry and Center for Computing in Engineering and Sciences, Universidade Estadual de Campinas (UNICAMP), Campinas, SP 13083-852, Brazil

## Abstract

We present PDBTools.jl, a lightweight and high-performance Julia package for reading, writing, selecting, and analyzing molecular structure data stored in PDB and mmCIF (PDBx) file formats. The package provides a compact and memory-efficient atom representation based on inline strings and single-precision floating-point coordinates, enabling the handling of very large structures on standard hardware. A flexible and customizable atom selection syntax inspired by VMD is augmented by native support for arbitrary Julia functions as selectors, offering exceptional expressiveness and performance for dynamic queries. Beyond file I/O and selection, PDBTools.jl includes high-performance implementations of key structural analysis algorithms: solvent-accessible surface area (SASA) calculation via the Shrake–Rupley method with Fibonacci lattice sampling, hydrogen bond detection, contact and distance maps, backbone dihedral angles for Ramachandran analysis, secondary structure assignment through integration with STRIDE and DSSP, and protein transfer free energy (*m*-value) calculations using the Tanford additive model. Cell list-based neighbor finding ensures O­(N) scaling for distance-dependent operations with support for periodic boundary conditions. PDBTools.jl is designed for molecular dynamics simulation workflows and integrates with Chemfiles.jl, MolSimToolkit.jl, and ComplexMixtures.jl. The package is freely available under the MIT license from the Julia General Registry, and full documentation can be found at https://m3g.github.io/PDBTools.jl.

## Graphical abstract

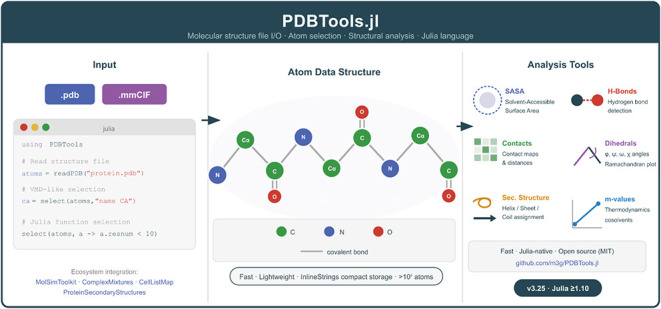

## Introduction

Structural biology and molecular dynamics (MD) simulations generate large volumes of molecular structure data, predominantly stored in the Protein Data Bank (PDB) format and the newer mmCIF (macromolecular crystallographic information file, also known as PDBx) format.^1^ The ability to read, write, filter, and analyze these files programmatically is a fundamental requirement for nearly every computational workflow in structural and computational chemistry, ranging from simple coordinate extraction to complex analyses of protein stability, ligand binding, and conformational dynamics.

Several well-established libraries exist for this purpose in Python, most notably Biopython^2^ and MDAnalysis.^2,3^ Within the Julia ecosystem, BioStructures.jl^4^ provides comprehensive support for PDB and mmCIF formats and is the primary tool for structural biology applications requiring strict format compliance. However, the MD simulation community often requires tools optimized for rapid, flexible analysis rather than crystallographic metadata management. In particular, operations performed repeatedly across thousands of simulation frames demand low-overhead data structures and fast algorithms.

The Julia programming language^5^ offers a compelling combination of high performance approaching that of compiled languages such as C and Fortran with the expressiveness and interactivity of high-level scripting languages. This makes Julia particularly attractive for scientific computing tasks where both development speed and execution performance are critical. Julia’s multiple dispatch system and its support for parametric types enable the creation of generic yet efficient data structures that can be extended without performance penalty.

PDBTools.jl was developed to fill the gap between lightweight file I/O and the need for practical, high-performance structural analysis in MD simulation workflows in Julia. The package deliberately prioritizes flexibility and speed over strict format compliance, providing tools that are immediately useful for the most common tasks in MD analysis: loading structures, selecting atoms, computing surfaces, detecting hydrogen bonds, and estimating thermodynamic quantities derived from structural properties. Here, we describe the design, key features, and performance characteristics of PDBTools.jl, currently in version v3.25.0.

## Software Description

### Atom Data Structure

The central data structure in PDBTools.jl is the *Atom­{T}* type, a mutable parametric struct that holds the complete information for a single atom as it would appear in a PDB record. Each atom stores its sequential index, the index as written in the PDB file, atom name, residue name, chain identifier, residue number (from the PDB file), sequential residue number, Cartesian coordinates (*x*, *y*, *z*), occupancy, temperature factor (B-factor), model number, segment name, element symbol, formal charge, and an optional user-defined field of generic type *T*. An internal flag field supports efficient processing during analysis.

Two design decisions are central to the memory efficiency of the data structure. First, all string fields (atom name, residue name, chain, segment name, and element) are stored as fixed-width string values (from InlineStrings.jl^6^), which are by default 15-character strings stored directly within the struct rather than as heap-allocated objects. This eliminates a large number of pointer indirections and greatly reduces the garbage collection pressure when processing files containing millions of atoms. Second, atomic coordinates are stored as Float32 values rather than Float64, halving the memory footprint of coordinate data with a negligible loss of precision for MD analysis purposes. The resulting Atom struct occupies approximately 144 bytes and is allocated once at construction, yielding efficient vector-of-struct layouts that are cache-friendly for sequential access patterns.

The type parameter *T* allows users to attach arbitrary per-atom data without modifying the core data structures. For example, partial charges or custom force-field parameters can be stored in the custom field using the *add_custom_field* function, and this augmented *Atom­{Float64}* type participates fully in all PDBTools operations through Julia’s multiple dispatch system. Any data type can be stored in a custom field, and multiple-dispatch can be used to define custom operations on the derived types.

### File Input and Output

PDBTools.jl supports reading and writing files in both PDB and mmCIF formats through the *read_pdb*, *read_mmcif*, *write_pdb*, and *write_mmcif* functions. File reading is performed with a flexible parser that tolerates common deviations from strict format specifications, which is important in practice because many PDB files produced by MD simulation software do not fully conform to the official PDB standard. An optional atom selection string or function can be supplied directly to the reading functions, so that only the atoms of interest are loaded into memory, a valuable feature when working with very large structures or trajectories.

Structures can also be retrieved directly from the Protein Data Bank using the *wget* function, which downloads a specified PDB entry in either the PDB or mmCIF format and returns the parsed atom vectors. This enables fully self-contained analysis scripts that do not require predownloaded structure files.

### Atom Selection

One of the most frequently used operations in structural analysis is the selection of subsets of atoms according to structural or chemical criteria. PDBTools.jl provides two complementary approaches to atom selection. The first is a declarative selection syntax inspired by VMD,^7^ supporting Boolean operators (*and*, *or*, *not*), comparison operators, parenthesization, and range expressions. A comprehensive set of keywords covers all standard atom attributes (name, resname, chain, residue number, coordinates, B-factor, occupancy, segment name, element, and model) as well as biochemical class macros such as protein, backbone, side chain, water, aromatic, charged, polar, nonpolar, hydrophobic, acidic, and basic. The selection syntax can be extended by the user with the definition of new keywords and macros.

The second approach allows any Julia function that accepts an *Atom* and returns a Boolean to be used directly as a selector. This provides full access to Julia’s expressiveness, including closures, custom predicates, and calls to external libraries. It also avoids the overhead of string parsing in performance-critical loops. The Select type acts as a callable wrapper that makes selection strings compatible with standard Julia higher order functions such as *filter*, *findall*, *findfirst*, and *findlast*, enabling idiomatic Julia workflows. An example of the selection syntax in action is shown in Figure 1.

**Figure 1:**
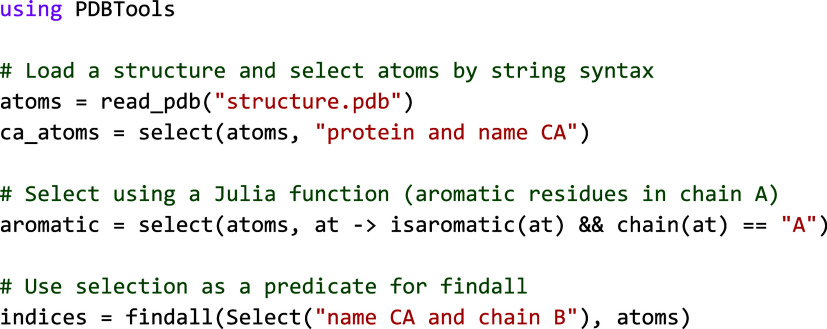
Example of the selection syntax of PDBTools.jl, using selection strings or regular Julia functions.

### Structural Hierarchy and Lazy Iterators

Molecular structures are inherently hierarchical: atoms belong to residues, residues belong to chains, chains belong to segments, and multiple conformations are organized as models. PDBTools.jl provides lazy iterators, *eachresidue*, *eachchain*, *eachsegment*, and *eachmodel*, that traverse an atom vector and yield lightweight view objects for each structural unit. These views contain only the index range into the underlying atom array and do not copy data, so iteration over residues or chains of a large protein is both memory-efficient and fast. Modifications made to atoms through these views directly affect the original vector. Convenience functions are provided for extracting properties at the residue level, such as residue name, sequence number, and chain identifier.

## Structural Analysis Tools

### Solvent-Accessible Surface Area

Solvent-accessible surface area (SASA) is one of the most widely used descriptors in structural analysis and is central to models of protein solvation, stability, and binding. PDBTools.jl implements the Shrake–Rupley rolling sphere algorithm^8^ using a Fibonacci lattice to generate a uniform distribution of sampling points on the surface of each atom. The neighbor search required to identify surface-occluding atoms is performed using CellListMap.jl,^9^ a cell list algorithm that scales as O­(N) with the number of atoms. The main entry point is the *sasa_particles* function, which returns a SASA object containing the accessible area per atom. The companion *sasa* function extracts the total or partial surfaces for any atom subset specified by the standard selection syntax. Notably, periodic boundary conditions are supported, making the function directly applicable to simulation boxes or the analysis of crystallographic packing effects on the accessible surface of biomolecules. The syntax for computing SASAs is illustrated in Figure 2.

**Figure 2:**
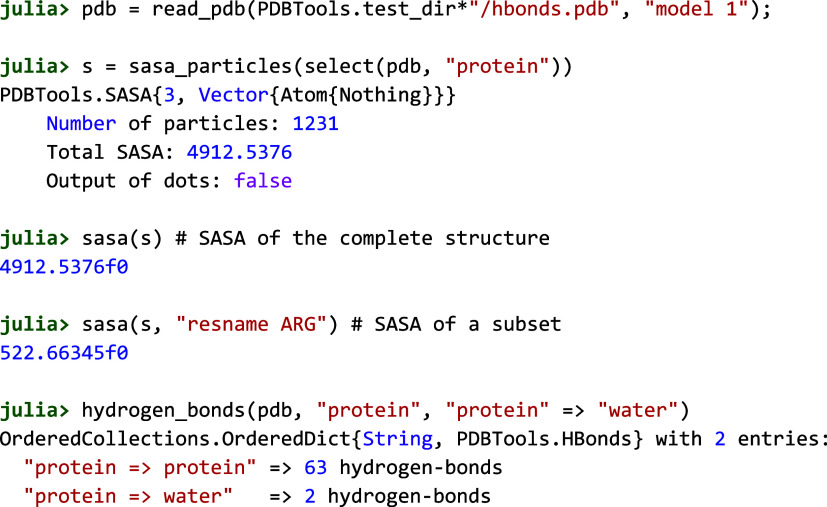
Computing the solvent-accessible surface area of a structure and a subset of the structure and hydrogen bond analysis.

### Hydrogen Bond Detection

Hydrogen bonds are fundamental determinants of the protein structure, stability, and recognition. The *hydrogen_bonds* function identifies hydrogen bond donor–hydrogen–acceptor triplets in a structure or between pairs of atom groups, applying standard geometric criteria: a maximum donor–acceptor distance (default 3.4 Å) and a maximum hydrogen–donor–acceptor angle (default 30°). Neighbor finding is again delegated to CellListMap.jl, yielding O­(N) performance. Multiple selection pairs can be specified in a single call; for example, intraprotein hydrogen bonds and protein–water hydrogen bonds can be computed simultaneously and returned in a dictionary keyed by the selection pair string, as shown in Figure 2. The resulting *HBonds* object is a full Julia iterable supporting *filter*, *findall*, indexing, and enumeration, enabling flexible downstream analysis of bond geometry and distribution.

### Contact and Distance Maps

Contact maps provide a compact representation of inter-residue spatial relationships and are widely used to characterize the protein fold topology and binding interfaces. The *contact_map* function computes either a discrete (binary contact) or a continuous (minimum distance) map for a single structure or between two chains. The minimum distance between all heavy atoms of each residue pair is computed, and the result is stored as a sparse matrix to minimize the memory usage. A gap parameter allows short-range sequence neighbors to be excluded, which is useful for focusing on the tertiary contacts. Arithmetic operations on *ContactMap* objects of the same type (subtraction and addition) enable a direct comparison of contact patterns between different conformations or models. Integration with Plots.jl^10^ provides a one-line heatmap visualization.

### Backbone Dihedral Angles and Ramachandran Analysis

Backbone dihedral angles φ (phi) and ψ (psi) are key descriptors of the protein secondary structure and are used in Ramachandran analysis to validate protein geometry and monitor conformational changes during simulations. The Ramachandran constructor iterates over all residues of a protein and computes the φ and ψ angles, returning a structured object that can be directly plotted as a Ramachandran diagram using the Plots.jl extension.

### Secondary Structure Assignment

Secondary structure assignment is provided through integration with the ProteinSecondaryStructures.jl package, which wraps the STRIDE^11^ and DSSP^12^ algorithms. The *stride_run* and *dssp_run* functions accept a PDBTools atom vector, write a temporary PDB file, invoke the external program, and parse the output, annotating each residue with its secondary-structure class. The result integrates naturally with the residue iteration interface described above.

### Protein Transfer Free Energy and *m*-Values

The effect of cosolvents on protein stability is frequently quantified through the *m*-value, defined as the derivative of the protein stability change with respect to cosolvent concentration.^13^ PDBTools.jl implements the Tanford additive transfer model in two parametrizations: the Auton–Bolen model^14,15^ and the Moeser–Horinek model.^16^ Given the SASA of two structural states, for example, a native and a denatured conformation, or a protein in bound and unbound forms, the *m*-value function computes the total, backbone, and side-chain contributions to the *m*-value, as well as per-residue decompositions. These per-residue contributions can be directly stored in the B-factor field of the atoms and visualized on the protein structure using standard molecular graphics software. Estimates for the denatured state SASA can be generated using the Creamer random coil model,^17,18^ enabling unfolding *m*-values to be computed from a single native structure.

## Performance

Performance is a primary design concern for PDBTools.jl. The use of InlineStrings avoids heap allocation for string fields, reducing pressure on the garbage collector and improving cache utilization when iterating over large atom vectors. The compact memory layout of the Atom struct (approximately 144 bytes) means that a 100,000-atom system occupies roughly 14 MB of RAM and a 1,000,000-atom system requires approximately 140 MB, well within the capacity of standard workstations.

Distance-dependent operations, SASA, hydrogen bond detection, and contact map computation, all rely on the CellListMap.jl library,^9^ which implements a cell list algorithm with O­(N) scaling in the number of atoms and optional multithreading. Figure 3 shows a comparison of the performance of PDBTools.jl with that of popular alternatives.^2,7,19,20^ PDBTools.jl provides state-of-the-art performance and linear scaling in all cases. It is noteworthy that the SASA implementation additionally employs SIMD (Single Instruction, Multiple Data) vectorization via the SIMD.jl package for the inner loop that tests whether surface dots are exposed or occluded by neighboring atoms. Together, these optimizations make the PDBTools.jl SASA implementation competitive with RustSASA,^21^ a very optimized library. RustSASA and PDBTools.jl provide parallel implementations. RustSASA can deliver ∼2–3× speedups using 4 to 10 threads, while currently, the PDBTools.jl implementation provides ∼1.5–2× speedups with the same resources but scales poorly for larger (>10k atoms) models. The benchmarks in Figure 3 were executed single-threaded in a 13th Gen Intel Core i7-13700KF CPU computer with 32GB of RAM and a NVIDIA GeForce RTX 4060 Ti graphic card. VMD is the only package that uses the GPU in these computations. Additional details on software versions, benchmark protocol, structural data sets, and analysis parameters are provided as a Zenodo repository under DOI 10.5281/zenodo.20030089.

**Figure 3:**
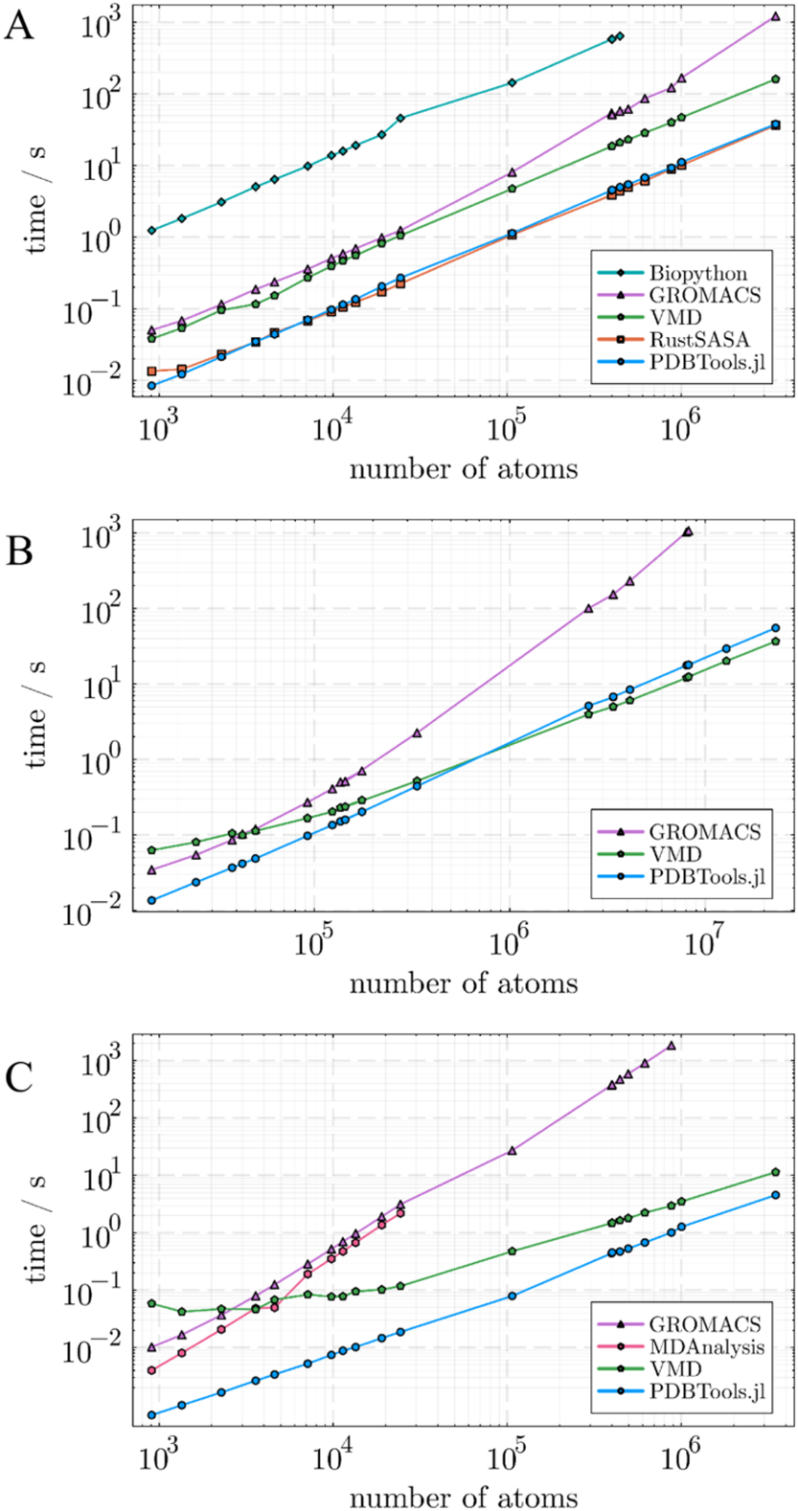
Performance of PDBTools.jl in comparison with popular alternatives for the computation of (A) solvent-accessible surface area; (B) hydrogen bonds; and (C) contact maps. PDBTools.jl provides state-of-the-art performance in all cases over a wide range of system sizes.

The atom selection engine compiles string-based selection expressions into Julia functions at parse time so that repeated application of the same selection, common in trajectory analysis loops, incurs no repeated parsing overhead. Users who require maximum performance in tight loops can supply compiled Julia functions directly, completely bypassing the selection parser.

## Integration with the Julia MD Ecosystem

PDBTools.jl is designed as a component of a broader Julia ecosystem for molecular dynamics simulation analysis. It serves as the primary structure-handling layer for MolSimToolkit.jl, a package that provides higher level tools for trajectory analysis including frame-by-frame iteration, geometric transformations, and thermodynamic property calculations. Similarly, ComplexMixtures.jl, a package for the analysis of preferential interactions and solvation structure using the Kirkwood–Buff theory,^22^ uses PDBTools.jl for all atom selection and structure reading operations.

This integration means that users who install either MolSimToolkit.jl or ComplexMixtures.jl automatically have access to the PDBTools.jl functionality, and analyses involving PDB file handling, atom selection, and SASA computation can be seamlessly combined with trajectory analysis and thermodynamic calculations in a unified Julia environment.

PDBTools.jl also provides optional integration with VMD through the *select_with_vmd* function, which allows for the full VMD selection syntax and all VMD-supported expressions and macros to be used from Julia. This is particularly valuable for users migrating existing VMD-based workflows to Julia, as it allows the gradual adoption of PDBTools.jl without requiring immediate translation of all selection expressions.

The analysis of MD trajectories can be performed by combining PDBTools.jl with Chemfiles.jl,^23^ which provides functionality for reading the most common MD trajectory formats. MolSimToolkit.jl provides a higher level of abstraction on top of Chemfiles.jl and integrates many of the analysis functions derived from PDBTools.jl. In Figure 4, we illustrate the computation of the SASA of a protein throughout an MD trajectory using Chemfiles and, alternatively, using the MolSimToolkit.jl implementation. The examples illustrate how simple it is to define functions that extend the computation of a property from a single structure to an MD trajectory analysis. In fact, other properties, such as secondary structure computation, coordination numbers, and hydrogen bond analysis, are already fully integrated into MolSimToolkit.jl.

**Figure 4:**
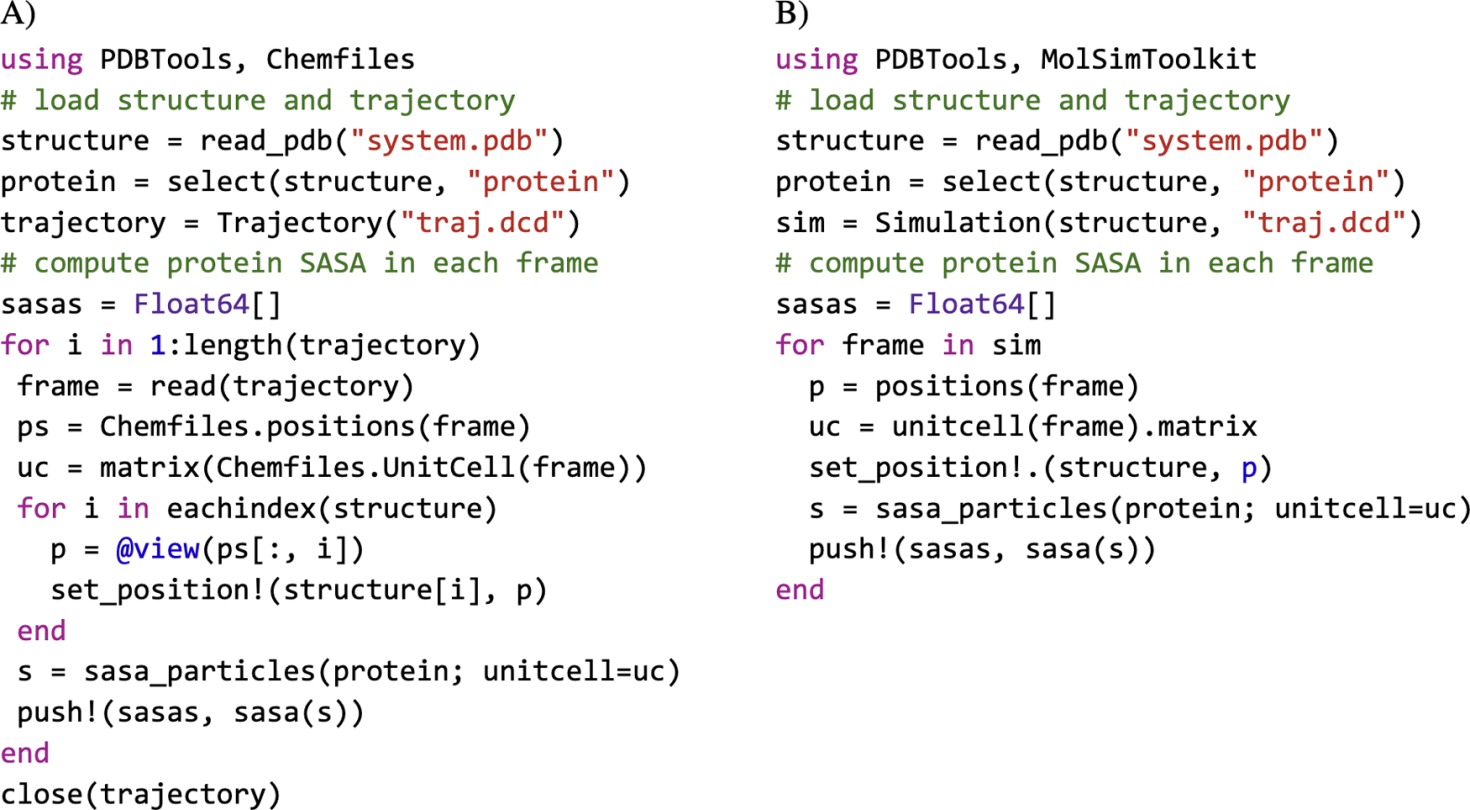
Implementation of a protein SASA calculation throughout a MD trajectory, by combining PDBTools.jl functions with (A) Chemfiles.jl and (B) MolSimToolkit.jl. In both cases, because the protein object is a view of the complete structure, updating the coordinates of the structure allows the direct recalculation of the protein SASA.

## Comparison with Related Software

BioStructures.jl^4^ is the most complete Julia package for PDB/mmCIF file handling and provides extensive support for crystallographic metadata, alternative conformations, and strict format validation. PDBTools.jl complements BioStructures.jl by prioritizing performance and flexibility over format completeness. Users who require comprehensive crystallographic metadata should prefer BioStructures.jl; users who need fast, flexible analysis of MD simulation structures might find PDBTools.jl to be better suited to their workflows. Notably, some features originally implemented for PDBTools.jl, such as the selection syntax and secondary structure calculations, were ported to BioStructures.jl for the benefit of the general community. Thus, it is expected that developments within the highly integrated Julia ecosystem will benefit all packages.

In the Python ecosystem, MDAnalysis^3^ and BioPython^2^ are the dominant tools for structure handling and MD analysis. MDAnalysis in particular provides extensive trajectory reading support for dozens of file formats, a feature that is outside the current scope of PDBTools.jl, which focuses on the structure-analysis layer. For trajectory analysis, PDBTools.jl users are directed to MolSimToolkit.jl. Compared to these Python packages, PDBTools.jl benefits from Julia’s just-in-time compilation and type specialization to achieve lower per-operation overhead, particularly for analysis functions such as SASA and hydrogen bond detection that are called many times in trajectory analysis loops. The implementation of custom analysis functions on top of the functionality provided by PDBTools.jl is advantageous because the package is written entirely in Julia, and novel tools can be implemented at a high level of language abstraction without sacrificing performance.

## Data and Software Availability

PDBTools.jl (currently in version 3.25.0) is free and open-source software distributed under an MIT license. The package is available from the Julia General Registry and can be installed in any Julia environment (version 1.10 or later) with
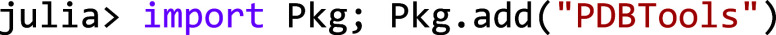


The source code, issue tracker, and contribution guidelines are hosted at https://github.com/m3g/PDBTools.jl. Comprehensive documentation, including function references, tutorials, and worked examples, is available at https://m3g.github.io/PDBTools.jl. The package is compatible with all major operating systems (Linux, macOS, and Windows) and requires no external dependencies beyond the Julia package ecosystem.

The data and scripts to reproduce the benchmarks in Figure 3 are available as a Zenodo repository under DOI: 10.5281/zenodo.20030089 (or through the link https://zenodo.org/records/20030089).

## Conclusions

PDBTools.jl provides a lightweight, flexible, and high-performance foundation for molecular structure file handling and analysis in the Julia programming language. By combining a compact, memory-efficient atom representation with a versatile selection interface and O­(N)-scaling implementations of key analysis algorithms, the package is well-suited to the demands of molecular dynamics simulation workflows. Its integration with the broader Julia MD analysis ecosystem and its compatibility with all major operating systems make it immediately useful for the computational chemistry and biophysics communities. We anticipate that PDBTools.jl will continue to evolve in response to user needs with ongoing development focused on additional analysis tools, improved structure handling support, and tighter integration with trajectory analysis pipelines. This package has been instrumental in many studies within our research group, for the analysis of the protein solvation structure and dynamics,^24,25^ polymer solutions,^26^ and very complex systems such as viral assemblies,^27^ and we hope that it will be useful for the general structural biology and molecular simulation communities.
